# Novel waste printed circuit board recycling process with molten salt

**DOI:** 10.1016/j.mex.2015.02.010

**Published:** 2015-02-21

**Authors:** Frank Riedewald, Maria Sousa-Gallagher

**Affiliations:** a Composite Recycling Ltd., The Rubicon Centre, CIT Campus, Bishopstown, Cork, Ireland; b Process and Chemical Engineering, School of Engineering, University College Cork, Ireland

**Keywords:** Printed circuit boards recycling, Float-sink separation, Metal recovery, Molten salt reactor

## Abstract

The objective of the method was to prove the concept of a novel waste PCBs recycling process which uses inert, stable molten salts as the direct heat transfer fluid and, simultaneously, uses this molten salt to separate the metal products in either liquid (solder, zinc, tin, lead, etc.) or solid (copper, gold, steel, palladium, etc.) form at the operating temperatures of 450–470 °C. The PCB recovery reactor is essentially a U-shaped reactor with the molten salt providing a continuous fluid, allowing molten salt access from different depths for metal recovery. A laboratory scale batch reactor was constructed using 316L as suitable construction material. For safety reasons, the inert, stable LiCl–KCl molten salts were used as direct heat transfer fluid. Recovered materials were washed with hot water to remove residual salt before metal recovery assessment. The impact of this work was to show metal separation using molten salts in one single unit, by using this novel reactor methodology.

• The reactor is a U-shaped reactor filled with a continuous liquid with a sloped bottom representing a novel reactor concept.
• This method uses large PCB pieces instead of shredded PCBs as the reactor volume is 2.2 L.
• The treated PCBs can be removed via leg B while the process is on-going.

## Graphical abstract

**Figure:**
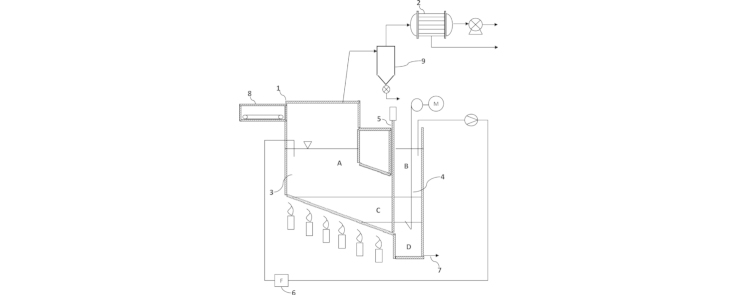

## Method details

### Description of the laboratory process

**Fig. 1:**
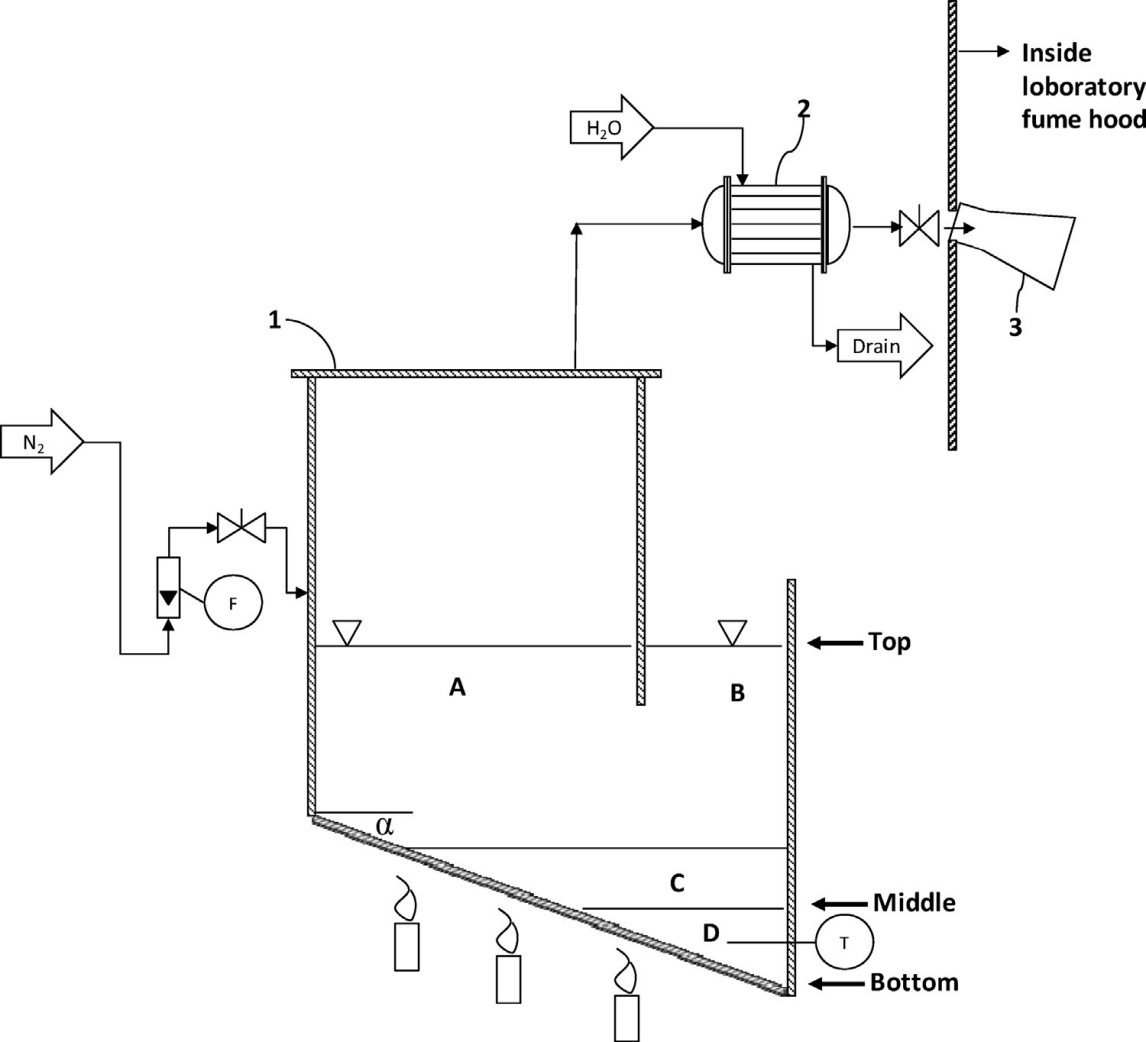
Layout and P&ID of the laboratory scale PCB separation/pyrolysis experiments (T = temperature gauge, F = nitrogen flow meter, N_2_ = nitrogen, *α* = 45 degrees, 1 = pyrolysis vessel, 2 = condenser, 3 = glass flask). Leg A and B: molten LiCl–KCl; area C: molten LiCl–KCl and glass and copper; area D: molten metals i.e., solder and heavy solid materials for example gold.

A P&ID of the laboratory scale waste PCB recycling process is presented in Fig. 1. The pyrolysis vessel, essentially a U-shaped reactor, was manufactured from a 6″ (125 mm) and a 4″ (100 mm) ANSI schedule 40, 316 L stainless steel pipe (6″ pipe = 7.1 mm wall thickness, 4″ pipe = 6.02 mm wall thickness) with a 7 mm 316L stainless steel plate welded onto these pipes as the bottom wall of pyrolysis vessel 1 which was sloped 45 degrees. Both legs of the pyrolysis vessel 1 could be opened; leg A by unbolting the top flange which ensures an airtight vessel, whereas leg B was open to the atmosphere allowing the operator to have access to the molten salt and the bottom of the molten salt reactor. The pyrolysis vapors generated by the pyrolysis reaction were condensed to pyrolysis oil by water-cooled condenser 2 and collected in glass beaker 3. The formation of an explosive atmosphere within the pyrolysis vessel 1 and downstream equipment during the experiments was prevented by a continuous nitrogen sweep inerting the pyrolysis vessel.

### Solder

**Fig. 2:**
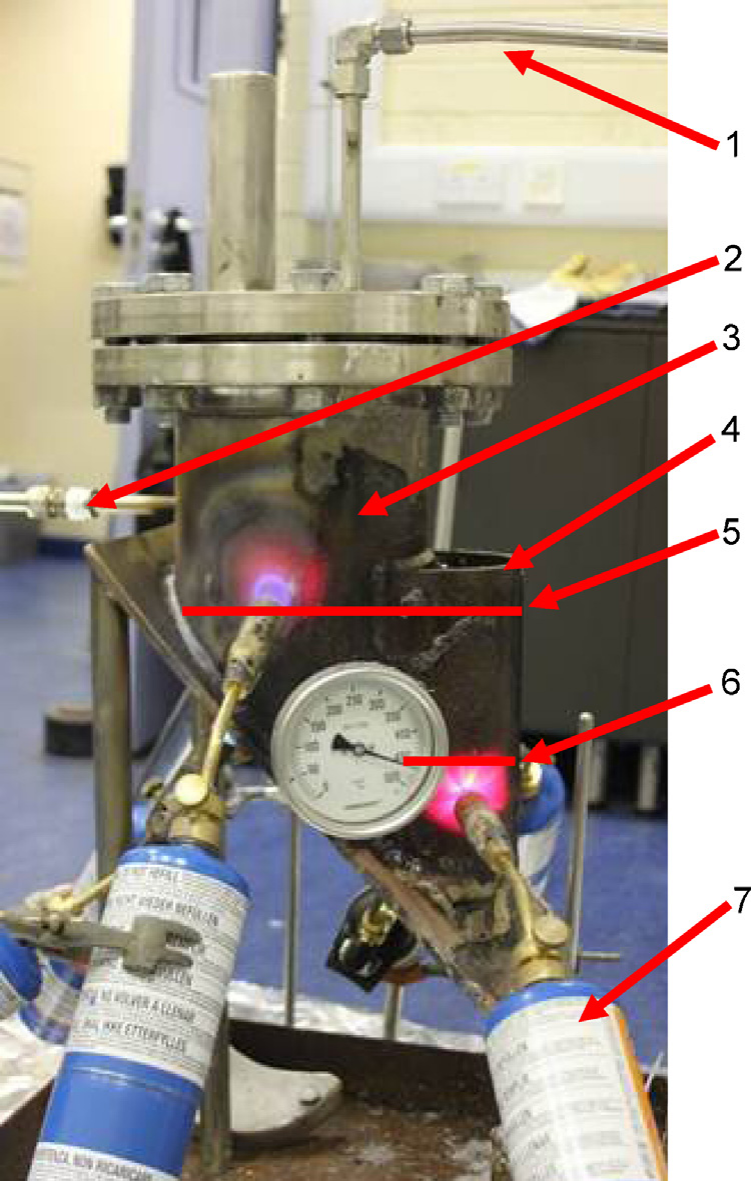
Image of the pyrolysis vessel with the temperature gauge showing 470 °C (1 = vent line of leg A, 2 = nitrogen supply line, 3 = leg A, 6″ or 125 mm pipe, 4 = leg B, 4″ or 100 mm pipe, 5 = level of molten salt, 6 = level of solder, 7 = blow torch).

1.5 kg of lead/tin (80/20wt%) commercial solder was added to the pyrolysis vessel to simulate a molten metal phase, which would accumulate in the full scale plant. The level of the molten metal was noted for the recovery phase of the solid materials for example stainless steel, which may separate to this level (Fig. 2).

### Molten salt

An eutectic mixture of technical grade lithium chloride (LiCl) and potassium chloride (KCl) salts (41.8 mol% KCl and 58.2 mol% LiCl (LiCl–KCl)) comprising of 2.2 L (3.5 kg of LiCl–KCl) was used to fill the remainder of the pyrolysis vessel. The physical properties of molten LiCl–KCl at a temperature of 450 °C are given in Table 1. As LiCl–KCl is stable and inert at the operating temperatures, it was chosen as the molten salt for the laboratory experiments.

Reactive salts i.e., molten salt oxidation (MSO) may also be used for PCB recycling. Flandineta et al. [1] used molten NaOH–KOH. For a full scale plant only nickel, a very expensive material and difficult to weld material, appears to be suitable to withstand the corrosive nature of molten NaOH–KOH [2]. But for molten LiCl–KCl, common 316L may be used as this salt was extensively tested for its potential use in molten salt nuclear reactors [3], [4].

### PCBs

PCBs from scrap personal computers (PCs) and televisions (TVs) were chosen in order to represent low and high value PCBs. The PCBs from the PCs were motherboards from PCs less than 2 years old, whereas the PCBs from the TVs were about 25 years old. The PCBs were cut to pieces of a maximum size of about 100–150 mm in order to fit into leg A. None of the components i.e., integrated circuits, transistors and switches were removed from the PCBs.

## Operational procedures

PCBs either from PCs or TVs were added to leg A. Leg A was closed and nitrogen sweep inerted at a flowrate of 10 Nm^3^/h for 5 min before the blow torches were switched on. Subsequently the nitrogen flow rate was reduced to about 1 Nm^3^/h. Once the pyrolysis temperature of 450–470 °C was reached, the temperature was held at that temperature until no more pyrolysis gases were visible to be emitted from the vessel into the glass beaker and then held for another half an hour. The reaction was deemed completed and leg A was opened for inspection i.e., to check for top dross or if the PCBs sank while at an operating temperature of 450–470 °C. This operating temperature was chosen as it is sufficiently high to melt the solder connecting the electronic components to the PCBs [5] and because it is higher than the resin decomposition temperature of about 380 °C [5]. Taking a safety factor to ensure a reasonable rate of resin destruction or pyrolysis, a minimum operating temperature of 450 °C was defined. Up to seven blow torches were used to provide the required temperature. The temperature of the molten salt was controlled by adjusting the torches. An Ashcroft temperature gauge (range of 0 to 500 °C, with a 1% ASME B40.3, grade A accuracy inserted into a thermowell manufactured from 316L stainless steel) was used to measure the temperature.

### Recovery of materials at operating temperature

The solids from the pyrolysis process of the PCBs may separate to four different locations depending on their density. They may separate to the top of the molten salt (see Fig. 1), they may separate to the middle i.e., to the interface between the molten salt and the molten metal, they may sink to the lowest part of the pyrolysis vessel and migrate to the bottom of leg B or they may be suspended within the molten salt phase (see also Table 1). Moreover some metals such as zinc or tin may form an alloy with the lead–tin solder phase at the bottom.

**Fig. 3:**
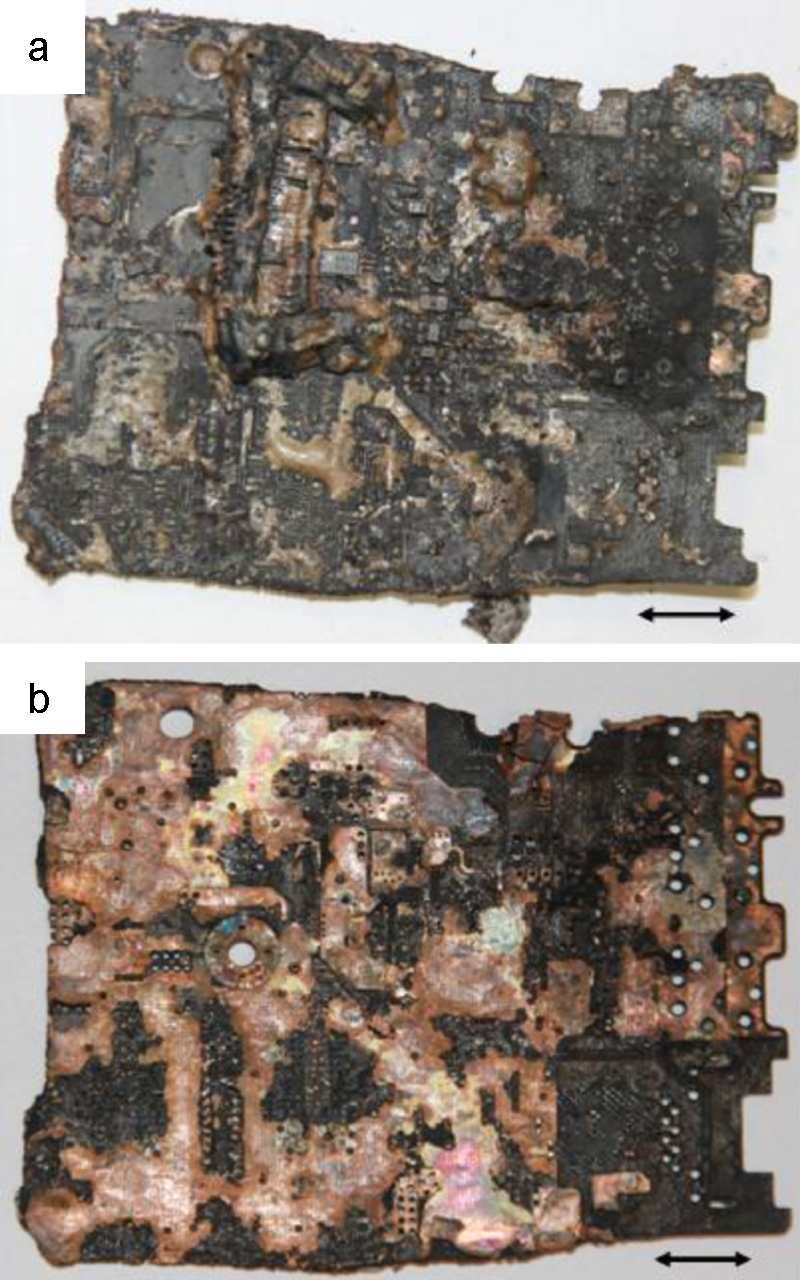
Recovered material from the middle of leg B before cleaning (a) and after cleaning (b) i.e., removing of residual salt and components showing a copper plate with some char (bar = 10 mm).

Thirty centimetre long carbon steel wires, which were bent to form a small hook at one end, were used to “fish” the solid materials from leg B. A motherboard as recovered from leg B is given in Fig. 3a. It shows that on removal from the molten salt, the motherboard is coated with salt and that some of the electrical components were still on the board. However after washing the salt off with 80 °C hot water, it was evident that the solder fixing the components to the board had melted off. As a consequence, the electrical components still on the board, were easily removed. The remaining PCB was essentially a thin copper plate as shown in Fig. 3b, which had lost most of its mechanical strength as the epoxy part of the composite was removed i.e., pyrolysed.

**Fig. 4:**
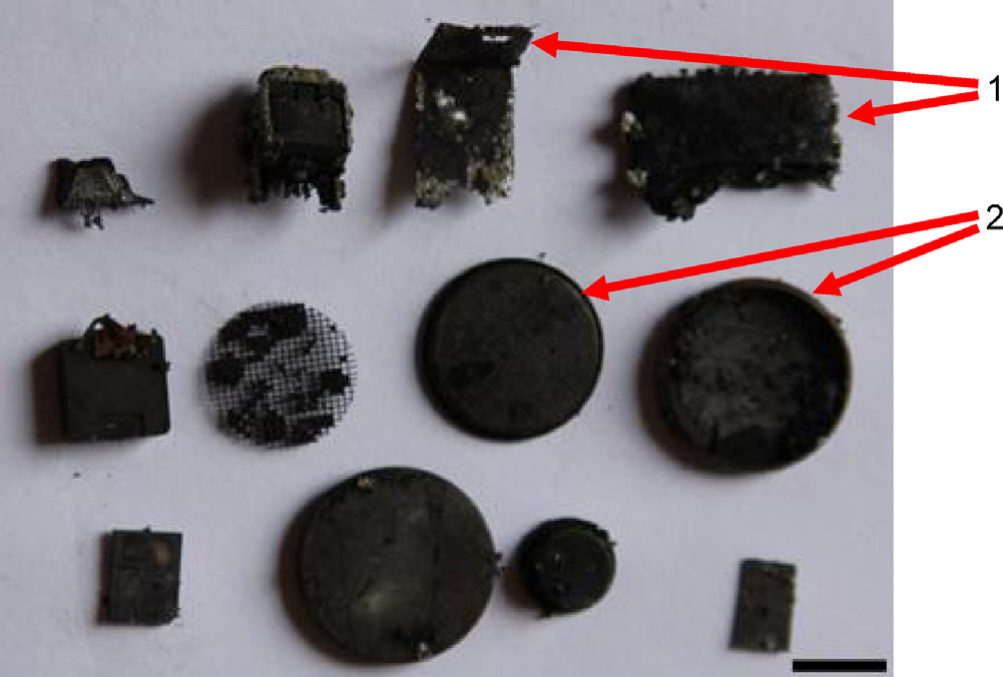
Example of other materials recovered from the middle of leg B (bar = 10 mm) 1 = metal parts from connectors; and other unidentified components from motherboards, 2 = stainless steel covers from button cell batteries.

After the motherboard was recovered, other materials were recovered also from the middle (Fig. 1) as shown in Fig. 4 such as the covers from button cell batteries, switches and other parts.

## Additional information

Every year about 400,000 tons of waste printed circuit boards (PCBs) are generated in Europe, but only about 15% of the scrap PCBs are subject to any kind of recycling [6]. PCBs are used to mechanically support and electrically connect electronic components using conductive pathways and are used in almost all electronic equipment such as televisions, computers or mobile phones [5]. As a composite waste comprised of resin, ceramics or glass, paper and metals, PCBs are difficult to recycle, but the metal content of PCBs makes PCBs a potentially large resource of precious and other metals [7]. On average PCBs contain copper, silver, gold, palladium in higher concentrations than their respective ores [6].

Pyrolysis is a process with great potential as it offers the possibility capable of recycling the metals present in PCBs in a self-sustaining by utilising the heat of combustion of the pyrolysis gases [8], [9], [10], [11]. The yield of the various products of waste PCBs pyrolysis such as metals, glass, oil and pyrolysis gas has already been investigated by various papers [8], [9], [10], [11]. Typical pyrolysis products of PCBs are on average of ∼70 wt% solids i.e., metals or glass, ∼23 wt% oil, and ∼6 wt% gas. This gas may be burned to self-sustain the PCB recycling process [12].

The full scale PCBs recycling process is envisaged to operate as follows: The waste PCBs are charged without mechanical pre-treatment that is intact into charging vessel 8 (see graphical abstract) after it was inerted. Then the PCBs are charged onto the molten salt 3 in pyrolysis vessel 1.

The pyrolysis vapors are continuously removed from the pyrolysis vessel 1. Solids entrained in the vapor stream are removed from the vapor stream by a cyclone 9 or by other means.

The light solids such as glass and some metals not molten at the operating temperature may collect in the pyrolysis vessel 1 above the collected metal i.e., in area C above level D from where this material is removed periodically by solids removal device 4. Such solid removal devices are used in the hot dip galvanising industry to remove heavy bottom phases from molten zinc baths and are hence available commercially [14]. Some of the light materials may have to be periodically drossed off after opening the pyrolysis vessel 1. Material suspended in the molten salt may be removed by filter 6.

The heavy compounds such as stainless steel, copper, gold or palladium but also metals molten at the operating temperature such as zinc, tin or lead separate to the bottom of the pyrolysis vessel 1 and, due to the slope of this wall, accumulate in area D where they may be drained off 7 or removed by solids removal device 4.

Gate 5 may be closed to ensure that the contact time of the PCBs with the pyrolysis liquid 3 is sufficiently long. Significant amounts of VOCs are not emitted from leg B as the molten salt 3 acts as a barrier sealing leg A from leg B. More details on the proposed full scale PCB treatment plant are provided in [15]. In summary, this PCB recycling process is a metal concentration process representing a new approach to PCB recycling.

The pyrolysis of PCBs containing brominated flame retardants may result in the generation of brominated organic compounds including potentially brominated dioxins and furans [9], [13]. As a result, a full scale waste PCB treatment system may require an air emission after treatment system i.e., scrubbing of HBr to comply with the relevant air emission regulations [16]. However such treatment systems are state-of-the-art [17] and therefore present no particular challenge. But further research is required to ascertain the maximum amount of HBr and/or brominated organics generated.

This laboratory scale experiment is a high temperature experiment involving molten metals and molten salts. The safety aspects of executing such an experiment should not be underestimated [18]. Some parts of this research were presented at Pyro 2014 [19].

## Figures and Tables

**Table 1:** Relevant physical properties of molten LiCl–KCl, metals and glass present in PCBs at 450 °C compiled from [20], [21], [22]. As a comparison, the properties of water at 25 °C are also given.

Compound	Density (kg/m^3^)	Surface tension (N/m)	Viscosity (Pa s)	Melting point (°C)	Vapor pressure (Pa)
LiCl–KCl at 450 °C (molten)	∼1,600	∼0.13	0.00146	355	133 at 800 °C
Glass at 450 °C (solid)	2400–2800	N/A	N/A	∼1000	Negligible
Zinc at 450 °C (molten)	6570	0.755	0.003254	419	100 at 477 °C
Steel at 450 °C (solid)	7750–8050	N/A	N/A	1425–1510	Negligible
Copper at 450 °C (solid)	8960	N/A	N/A	1084	Negligible
Lead at 450 °C (molten)	10,660	0.46	0.00206	327.5	1 at 705 °C
Palladium at 450 °C (solid)	12,160	N/A	N/A	1551	Negligible
Gold at 450 °C (solid)	19,320	N/A	N/A	1063	Negligible
Water 25 °C	1000	0.072	0.001003	0	3000
